# Hollow boron nitride nanospheres as boron reservoir for prostate cancer treatment

**DOI:** 10.1038/ncomms13936

**Published:** 2017-01-06

**Authors:** Xia Li, Xiupeng Wang, Jun Zhang, Nobutaka Hanagata, Xuebin Wang, Qunhong Weng, Atsuo Ito, Yoshio Bando, Dmitri Golberg

**Affiliations:** 1 World Premier International Center for Materials Nanoarchitectonics (WPI-MANA), National Institute for Materials Science (NIMS), Namiki 1-1, Tsukuba, Ibaraki 305-0044, Japan; 2 Health Research Institute, Department of Life Science and Biotechnology, National Institute of Advanced Industrial Science and Technology (AIST), Central 6, 1-1-1 Higashi, Tsukuba, Ibaraki 305-8566, Japan; 3 School of Materials Science and Engineering, Hebei Key Laboratory of Boron Nitride Micro and Nano Materials, Hebei University of Technology, Tianjin 300130, China; 4 Nanotechnology Innovation Station, National Institute for Materials Science (NIMS), 1-2-1 Sengen, Tsukuba, 305-0047, Japan

## Abstract

High global incidence of prostate cancer has led to a focus on prevention and treatment strategies to reduce the impact of this disease in public health. Boron compounds are increasingly recognized as preventative and chemotherapeutic agents. However, systemic administration of soluble boron compounds is hampered by their short half-life and low effectiveness. Here we report on hollow boron nitride (BN) spheres with controlled crystallinity and boron release that decrease cell viability and increase prostate cancer cell apoptosis. *In vivo* experiments on subcutaneous tumour mouse models treated with BN spheres demonstrated significant suppression of tumour growth. An orthotopic tumour growth model was also utilized and further confirmed the in vivo anti-cancer efficacy of BN spheres. Moreover, the administration of hollow BN spheres with paclitaxel leads to synergetic effects in the suppression of tumour growth. The work demonstrates that hollow BN spheres may function as a new agent for prostate cancer treatment.

## Abstract (web summary)

Use of soluble boron compounds in prostate cancer therapy is hampered by their short half-life time and low effectiveness. Here, the authors show that boron nitride nanospheres with controlled boron release can reduce proliferation of prostate cancer cells and inhibit tumour growth in animal models.

Prostate cancer is one of the most common cancers for males, particularly in the developed Western countries^1^. The high global incidence of prostate cancer has led to a focus on prevention and treatment strategies to reduce the public health impact of the disease^2^,^3^,^4^,^5^,^6^. For prostate cancer, the main clinical treatments include active surveillance, surgery^7^, radiation therapy^8^, hormone therapy^9^ and chemotherapy^10^ with drugs, such as paclitaxel (PTX) and docetaxel. An epidemiological study shows that boron (B) intake can reduce the risk of prostate cancer^11^,^12^ for human by up to 54% (ref. 13). Boric acid (BA), the dominant form of B in plasma, has been tested as a preventative and therapeutic agent against prostate cancer. For example, *in vitro* BA inhibits the proliferation of prostate cancer cells in a dose-dependent manner^14^. In the animal model, BA decreases serum prostate-specific antigen (PSA) levels by 88%, inhibits the LNCap tumour growth by 38% and reduces insulin-like growth factor-1 in nude mice injected with human LNCap prostate cancer cells^15^. Mechanisms of B-mediated anticancer action in prostate cancer include the reduction of intracellular calcium signals and storage, the decrease in enzymatic activities (serine protease, NAD-dehydrogenases and so on) and the inhibition of the cancer cell proliferation^14^,^16^,^17^,^18^,^19^.

Recently, B compounds have attracted attention as preventative and therapeutic agents for prostate cancer and other cancers^11^,^12^,^13^,^14^,^15^,^16^,^17^,^19^,^20^,^21^,^22^,^23^,^24^,^25^,^26^,^27^. However, systemic administration of soluble B compounds, such as BA, associates with the drawbacks of its short half-life period, low bioavailability, requirement of frequent administration, low fraction arrived in the tumour site and limited effectiveness for prostate cancer treatments. Moreover, the therapeutic window of BA for prostate cancer cells, which is ∼100 times higher than its average serum level in human, suggests difficulty in systemic administration of soluble B compounds without toxicity^14^,^21^,^23^. Local delivery of anticancer drug or agents in a sustained manner either to the region that contains a tumour or directly within the tumour has the advantage of increasing tumour exposure to drug while limiting systemic toxicity^28^,^29^. Therefore, local delivery of a sparsely soluble B-containing compound, as an alternative to soluble B compounds, might hold promise as a preventative and therapeutic agent for prostate cancer treatment. In addition, the systematic administration of a sparsely soluble B-containing compound in a nanoparticle format can facilitate the passive targeting of drugs into the tumour sites, decrease the side effects and enhance the antitumour efficacy with the aid of enhanced permeability and retention effects^30^. Typically, the sparsely soluble B-containing compound is boron nitride (BN) that is structurally analogous to carbon. To date, BN has been used as a delivery vehicle^31^,^32^,^33^ for anticancer drugs such as doxorubicin, similarly to other nano delivery vehicles, such as carbon nanotubes^34^, graphene^35^, mesoporous silica^36^, calcium phosphate^37^ and polymers^38^. However, there have been no reports regarding the effectiveness of BN itself in cancer treatments.

Herein we fabricated hollow BN spheres with controlled crystallinity and solubility to guide B release by adjusting the posttreatment temperature. Androgen-sensitive LNCap and androgen-independent DU-145 prostate cancer cell lines were used to evaluate the effects of hollow BN spheres on the apoptosis, necrosis and proliferation of the prostate cancer cells *in vitro*. The death mechanism of LNCap and DU145 prostate cancer cells treated with BA or BN spheres were evaluated and compared by a spectral cell analyzer. Then male BALB/c-nu/nu mice were used to confirm the effects of hollow BN spheres on the suppression of prostate cancer occurrence and inhibition of tumour growth *in vivo*.

## Results

### Physicochemical characterization of hollow BN spheres

**Figure 1:**
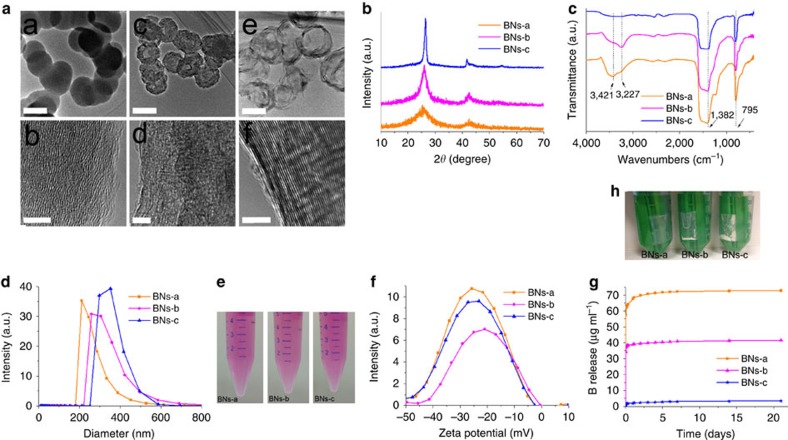
Physicochemical characterization of BN spheres. (**a**) Transmission electron microscopic images of BN nanospheres: BNs-a (a,b), BNs-b (c,d), and BNs-c (e,f). Scale bar: a,c,e, 200 nm; b,d,f, 5 nm. (**b**) Wide-angle X-ray diffraction patterns of the BN nanospheres. (**c**) FTIR patterns of the BN nanospheres. (**d**) Particle size distribution of the BN spheres in water. (**e**) Suspension of BN spheres in culture medium at 100 μg ml^−1^. (**f**) Zeta potential of BNs-a, -b and -c in PBS buffer. (**g**) Boron release for BNs-a, -b and -c in acetate buffer pH=4.6 at different time. (**h**) The residual particles after immersing same amount of BNs-a, -b and -c samples in acetate buffer for 1 month.

Hollow BN spheres were synthesized via the chemical vapour deposition (CVD) reaction of trimethoxyborane (B(OMe)_3_) using modified method developed previously^39^, in which the second-stage annealing process was conducted in Ar rather than in NH_3_ atmosphere. Transmission electron microscopic images prove the successful synthesis of BN nanospheres (Fig. 1a). The BNs-a sample shows a solid sphere structure with an approximate diameter of 200 nm and low crystallinity characterized by a long-range disorder. The BNs-b shows a hollow sphere structure with the same diameter and wall thickness of 50–60 nm. For BNs-c, a hollow nanostructure with a wall thickness of about 20 nm and high crystallinity characterized by the state of long-range order is observed.

The BN spheres are adjustable with respect to crystallinity by means of posttreatment temperature variations (Fig. 1b). As a whole, with an increase in posttreatment temperature, the crystallinity increased and the full width at half-maximum for the peaks in the wide-angle X-ray diffraction patterns becomes narrower. The BNs-a spheres exhibit an amorphous nature with a broad peak at around 26°. The BNs-b spheres are higher in crystallinity than BNs-a, exhibiting two crystalline peaks at around 26.4° and 42° that correspond to (002) and (100), respectively. The BNs-c spheres are the highest with regard to crystallinity, exhibiting a much narrower peak width for (002) and (100) reflections than those for BNs-b.

All the BN spheres show typical fourier transform infrared spectroscopy (FTIR) absorption bands of B-N stretching at 1,382 cm^−1^ and B-N-B bending at 795 cm^−1^ (Fig. 1c). The presence of hydroxyl groups is confirmed by the O-H stretching band at 3,421 cm^−1^ for BNs-a. A shoulder at 3,227 cm^−1^ indicates the asymmetric stretching of the N-H group. A weak band at around 1,200–1,250 cm^−1^ is attributed to B-N-O stretching. With an increase in calcination temperature from 900 to 1,025 °C for BNs-b, the O-H stretching band becomes weaker while the N-H stretching band becomes stronger. For BNs-c, calcined at 1,400 °C, both the O-H and N-H bands become very weak. X-ray photoelectron spectroscopy reveals the high content of oxygen (O) in BN spheres (Supplementary Fig. 1). As shown in Fig. 1d, the BNs-a, -b and -c nanospheres exhibit hydrodynamic diameters of 263.5±66.5, 334.1±87.8 and 351.8±56.3, respectively, which are slightly higher than those obtained from the transmission electron microscopic observations. In addition, unlike other BN materials such as BN nanotubes, all the BN spheres show good dispersibility in cell culture medium (Fig. 1e). The presence of large amount of hydroxyl groups (Fig. 1c) and a high content of oxygen (Supplementary Fig. 1) result in their high hydrophilicity and good dispersibility in an aqueous solution. At pH=7.4, the BNs-a, -b and -c spheres all show zeta potentials centred at around −21 to −25 mV (Fig. 1f).

### B release from hollow BN spheres

Release of B from BN spheres with controlled crystallinity kept in dialysis membrane bags was analysed in an acetate buffer at pH 4.6. BN spheres with high crystallinity show slow B release (Fig. 1g). BNs-a spheres with the lowest crystallinity show the highest B release speed, which is about 20 times that of BNs-c spheres and 2 times that of BNs-b spheres. Figure 1h displays the BN nanospheres remain in the dialysis membrane bags after immersing the same amount of BNs-a, -b and -c samples in the acetate buffer for 1 month. It can be seen that the BNs-a sample almost totally degrades, while the BNs-c sample remains in a large amount. Supplementary Fig. 2 shows the B release rate for different BN spheres under various conditions of pH, temperature and concentration. For all the BN spheres, the B release rate increases with the increase in temperature. In addition, the B release rate nearly linearly increases with the increase in the initial BN materials concentration. For BNs-b, pH value has a negligible effect on B release at a low temperature, whereas, at a high temperature, high pH value results in the increase in the B release. The dynamic studies of structural evolution for BN spheres are presented in Supplementary Fig. 3. For BNs-a sample, the spherical particle, around 200 nm in diameter, gradually degrades from the edge area at the initial stage and then transforms into smaller clusters, about 5–20 nm in diameter, 3 days later. For BNs-b sample, the hollow spherical particle degrades in a different way compared with the BNs-a sample. The defect sites in the wall gradually degrade to form a porous structure and the hollow spherical wall structures are still preserved after 3 days. After 10 days, the partial degradation in the edge area and a marginal amount of small clusters are observed. Supplementary Fig. 4 reveals FTIR spectra of the hydrolysed products of BN nanospheres. New absorption bands at 1,228 and 1,185 cm^−1^ are attributed to the B-O group for the BA and B-N-O groups, respectively. In addition, the obtained products possess new absorption bands at 1,096, 1,023, 916 and 689 cm^−1^, which are identified as ammonium borate hydrates. In contrast with Fig. 1b, wide-angle X-ray diffraction patterns of the hydrolysed products of BN spheres suggest the presence of BA and ammonium borate hydrates (Supplementary Fig. 5), which is consistent with the FTIR results.

### Cytotoxicity assay

Inhibitory effects of hollow BN spheres on proliferation and viability of androgen-sensitive LNCap and androgen-independent DU145 prostate cancer cell lines were assessed by the WST-8 method (Cell Counting Kit-8). Both BA and the BN spheres reduce cell viability in a dose-dependent manner (Supplementary Fig. 6). For LNCap prostate cancer cells, all the BN spheres decrease cell viability greater than BA. Among them, BNs-b with a moderate crystallinity and B release best inhibits LNCap cell viability. For example, after 3 days' exposure at 5 μg ml^−1^, BNs-b induces much lower cell viability than BA, BNs-a and BNs-c. In addition, at a higher concentration or longer exposure time, BNs-b as well as BNs-c induces much higher inhibition for LNCap cells than BA and BNs-a. There is also a tendency for BNs-b to inhibit best DU145 cell viability after 3-day exposure. Moreover, after 6 days' exposure at a concentration of up to 5 μg ml^−1^, BNs-b still induces the lowest DU145 cell viability. After long incubation for 6 days at a high concentration of 25 μg ml^−1^, BA, BNs-a and BNs-b decrease DU145 cell viability greatly, compared with BNs-c. As a whole, LNCap prostate cancer cells are more sensitive to BNs-b and -c with relatively higher crystallinity and slower B release, while DU145 prostate cancer cells are more sensitive to BA, BNs-a and BNs-b with the lower crystallinity and faster B release.

**Figure 2:**
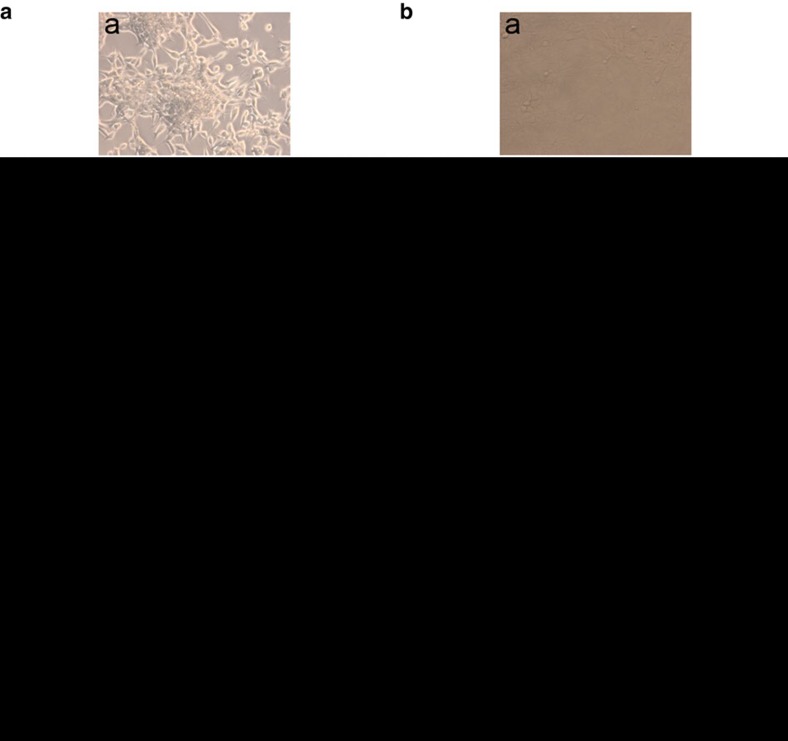
BN spheres alter cell morphology. (**a**,**b**) Optical microscopy images of (**a**) LNCap and (**b**) DU145 prostate cancer cells after exposure to original culture mediums (a) and culture medium containing different samples at 5 μg ml^−1^ (b: BA; d: BNs-a; f: BNs-b; h: BNs-c) and 25 μg ml^−1^ (c: BA; e: BNs-a; g: BNs-b; i: BNs-c) for 6 days. BA at the equivalent B concentration was used as control.

Light microscopic observation revealed significant morphological alterations between the cells treated with BN spheres and BA as shown in Fig. 2. After 6-day exposure of LNCap cancer cells to BA and BNs-a, there are no obvious changes in morphology. However, the presence of BNs-b results in a significant decrease in cell number and obvious aggregation. The presence of BNs-c also results in a decrease in cell number, although the trend is not obvious as for BNs-b. DU145 cells treated with BA, BNs-a and BNs-b for 6 days overall shrink and become smaller, showing a typical apoptosis process. Especially for BNs-b, the obvious aggregation of particles in the cytoplasm can be observed. However, for BNs-c, a large number of DU145 cells remain attached to the plate and only a small number of cells become smaller.

### Cell death mechanism induced by hollow BN spheres

**Figure 3:**
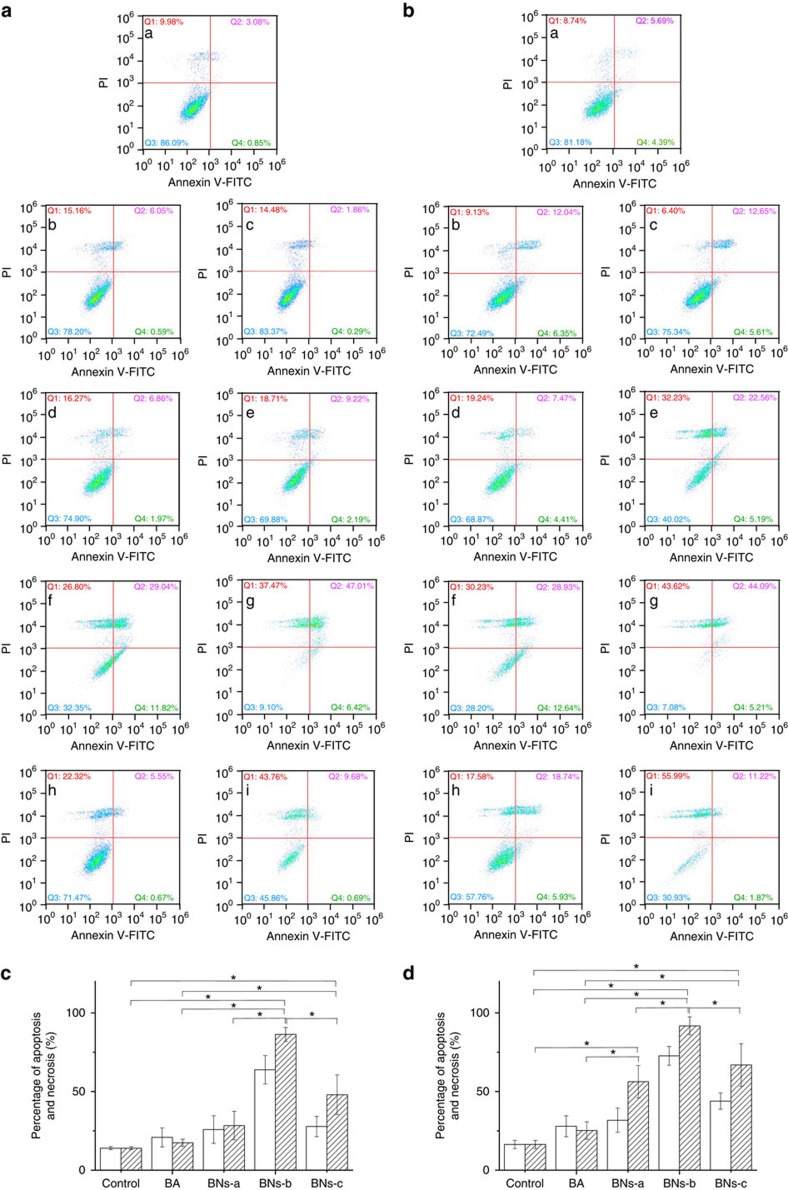
BN spheres induce apoptosis and necrosis in LNCap prostate cancer cells. (**a**,**b**) Evaluation of the death pathways of LNCap cells treated with BA or BN nanoparticles supplemented culture medium at BN concentration of 5 μg ml^−1^ (b: BA; d: BNs-a; f: BNs-b; h: BNs-c) and 25 μg ml^−1^ (c: BA; e: BNs-a; g: BNs-b; i: BNs-c) for 3 days (**a**) or 6 days (**b**). BA containing equivalent B was used for comparison. Culture medium was used as control (a). Q1, Q2, Q3 and Q4 zones represent necrosis, late apoptosis, normality and early apoptosis, respectively. (**c**,**d**) Statistical analysis of the percentage of apoptosis and necrosis in LNCap cells treated with BA or BN nanoparticles for 3 days (**c**) and 6 days (**d**). Blank: 5 μg ml^−1^; Slash: 25 μg ml^−1^. Data in **c**,**d** are shown as mean±s.d., ANOVA, *P<0.05, *n*=3.

**Figure 4:**
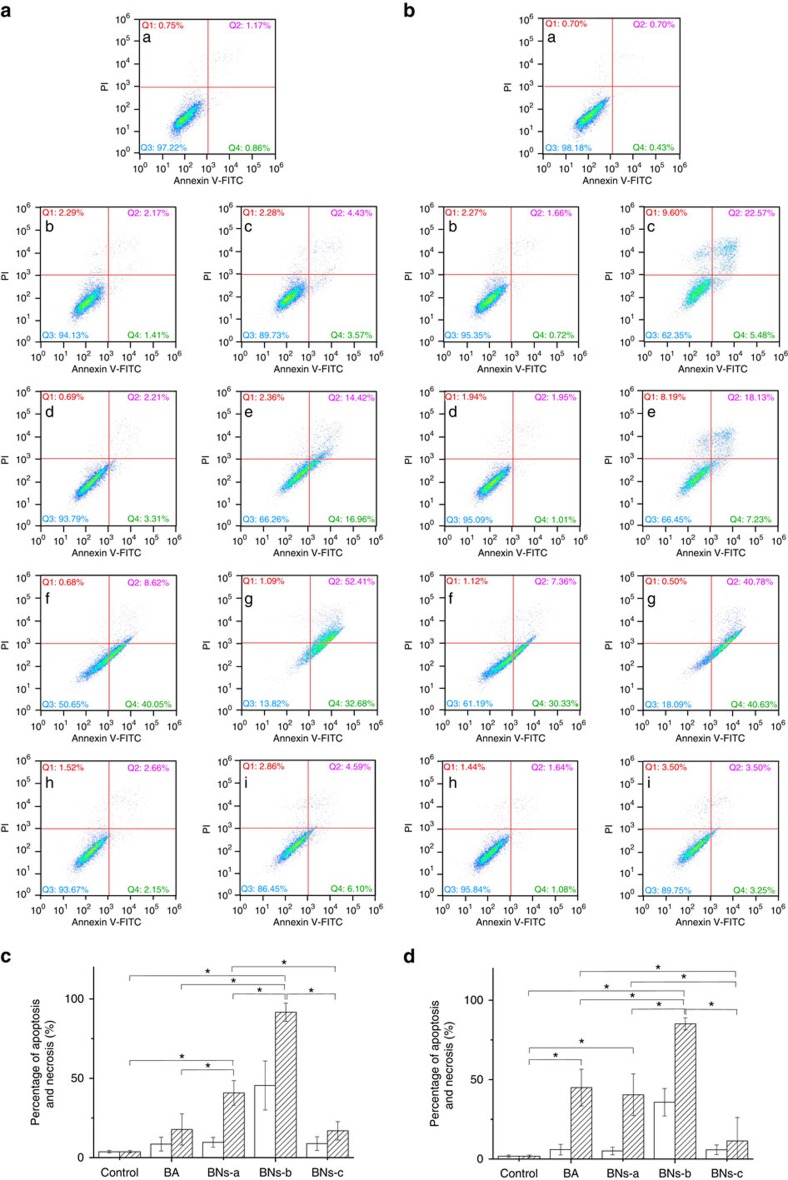
BN spheres induce apoptosis and necrosis in DU145 prostate cancer cells. (**a**,**b**) Evaluation of the death pathways of DU145 cells treated with BA or BN nanoparticles supplemented culture medium at BN concentration of 5 μg ml^−1^ (b: BA; d: BNs-a; f: BNs-b; h: BNs-c) and 25 μg ml^−1^ (c: BA; e: BNs-a; g: BNs-b; i: BNs-c) for 3 days (**a**) or 6 days (**b**). BA containing equivalent B was used for comparison. Culture medium was used as control (a). Q1, Q2, Q3 and Q4 zones represent necrosis, late apoptosis, normality and early apoptosis, respectively. (**c**,**d**) Statistical analysis of the percentage of apoptosis and necrosis in DU145 cells treated with BA or BN nanoparticles for 3 days (**c**) and 6 days (**d**). Blank: 5 μg ml^−1^; Slash: 25 μg ml^−1^. Data in **c**,**d** are shown as mean±s.d., ANOVA, *P<0.05, *n*=3.

LNCap and DU145 prostate cancer cells treated with BA or BN spheres were evaluated and compared by a spectral cell analyser (Figs 3 and 4). Cells stained annexin-V-FITC^+^/PI^−^ are considered as early apoptotic; cells stained annexin-V-FITC^+^/PI^+^ are considered as late apoptotic; cells stained annexin-V-FITC^−^/PI^+^ are considered as necrotic. Compared with blank control, all BN spheres exhibit obvious cytotoxicity against LNCap cells in a dose-dependent manner (Fig. 3c,d). In contrast, BA shows only weak cytotoxicity to LNCap cells regardless of dose. BNs-a enhances the fraction of early apoptosis, late apoptosis and necrosis, compared with BA (Fig. 3a,b). BNs-b best enhances the fraction of early apoptosis, late apoptosis and necrosis regardless of incubation time. For example, 3 days' exposure to 5 μg ml^−1^ of BNs-b is obviously cytotoxic, being characterized by 11.82% in early apoptosis, 29.04% in late apoptosis and 26.80% in cellular necrosis fractions (Fig. 3a(f)). When BNs-b dose is increased to 25 μg ml^−1^, the cytotoxicity increases to a level of 6.42% in early apoptosis, 47.01% in late apoptosis and 37.47% in necrosis (Fig. 3a(g)). However, exposure to BNs-c with high crystallinity and a very low rate of B release results in lower level of early apoptosis and late apoptosis than exposure to BNs-b. For example, 3 days' exposure to 25 μg ml^−1^ of BNs-c shows 0.69% in early apoptosis and 9.68% in late apoptosis (Fig. 3a(i)), which is much lower than exposure to BNs-b under the same conditions (6.42% and 47.01%, respectively). In addition, a decrease in solubility, thus an increase in crystallinity, clearly correlates with an increase in necrosis of LNCap cells: for example, the necrosis ratios for BA, BNs-a, -b and -c are about 14.48, 18.71, 37.47 and 43.76%, respectively, at 25 μg ml^−1^ for 3 days.

Similarly, BA and BN spheres exhibit obvious cytotoxicity against DU145 cells in a dose-dependent manner (Fig. 4c,d). For BA at a low concentration of 5 μg ml^−1^ or after a short period of 3 days, cytotoxicity against DU145 cells is weak. However, 6-days exposure to 25 μg ml^−1^ of BA is obviously cytotoxic, being characterized by 9.60% in cellular necrosis, 22.57% in late apoptosis and 5.48% in early apoptosis fractions (Fig. 4b(c)), which suggests that the cytotoxicity of BA is related to both the apoptosis and necrosis. Three days' exposure to 25 μg ml^−1^ of BNs-a enhances fractions of early (16.96%) and late (14.42%) apoptosis (Fig. 4a(e)), compared with that of BA (3.57% and 4.43%, respectively) (Fig. 4a(c)). Three days' exposure to BNs-b considerably enhances fractions of early apoptosis (40.05% at 5 μg ml^−1^; 32.68% at 25 μg ml^−1^) and late apoptosis (8.62% at 5 μg ml^−1^; 52.41% at 25 μg ml^−1^) (Fig. 4a(f,g)). After 6 days' incubation, BNs-b demonstrates similar results to those after 3 days. However, BNs-c shows much weaker effects on DU145 cells compared with BA and other two BN spheres regardless of incubation time.

**Figure 5:**
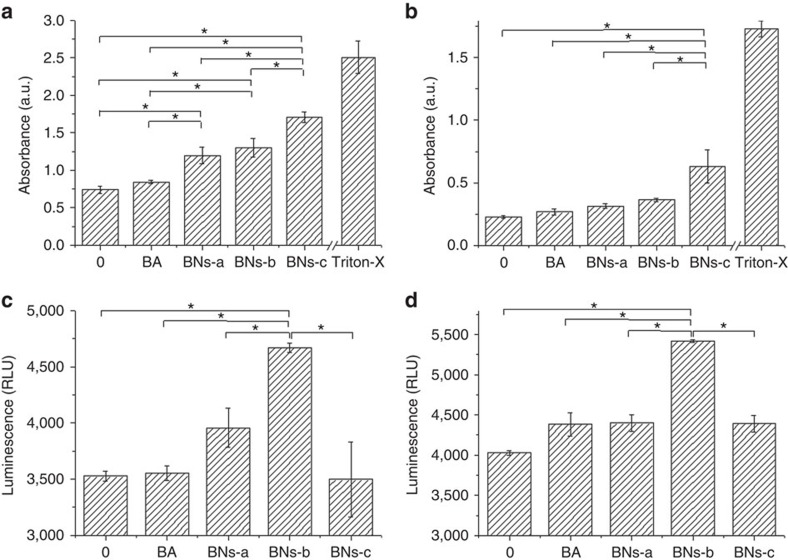
The levels of two key damage-associated molecular pattern protein biomarkers. LDH cytotoxicity for (**a**) LNCap and (**b**) DU145 prostate cancer cells after 16 h (*n*=4); Caspase-3/7 activity for (**c**) LNCap and (**d**) DU145 prostate cancer cells after 16 h (*n*=2). Data in **a**–**d** are shown as mean±s.d., ANOVA, *P<0.05.

Furthermore, the levels of two key damage-associated molecular pattern protein biomarkers, capase-3/7 and lactate dehydrogenase (LDH) release, were examined to evaluate apoptosis and necrosis, respectively (Fig. 5; Supplementary Figs 7 and 8). The results indicate that the apoptosis (capase-3/7) and the necrosis (LDH) are enhanced remarkably by BN spheres, compared with BA, further confirming the flow cytometric results. Both LNCap and DU145 prostate cancer cells show an increase in LDH release with the increase in crystallinity and decrease in solubility. It can be seen that the LDH release shows the following sequence: BNs-c>BNs-b>BNs-a>BA>control. As a whole, LNCap cells exhibit much higher LDH release than DU145 cells. With the addition of BA or BN spheres, caspase-3/7 contents increase. Among all the samples, BNs-b shows the highest caspase-3/7 activity both for LNCap and DU145 cells. Moreover, totally, DU145 cells exhibit higher caspase-3/7 activity than LNCap cells.

### *In vivo* anticancer effects of hollow BN spheres

**Figure 6:**
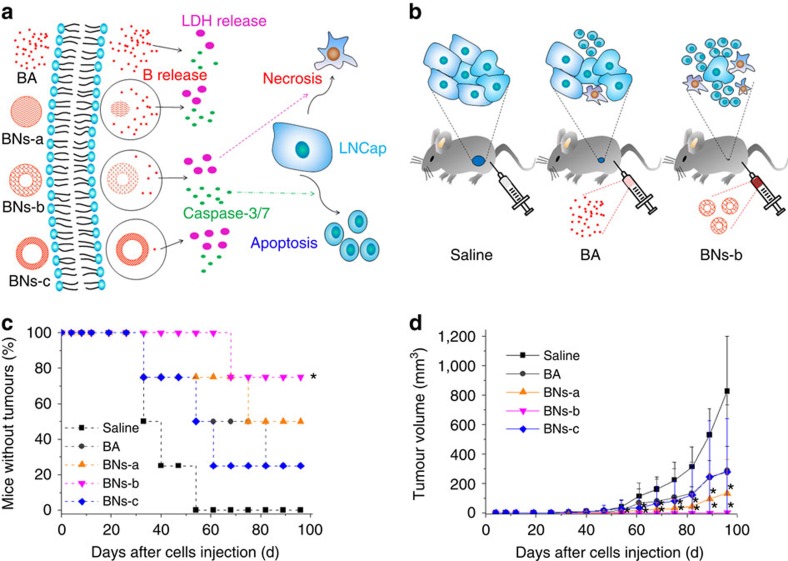
Effects of BN spheres on cellular and *in vivo* subcutaneously injected prostate cancer models. (**a**) BA or hollow BN spheres with controlled B release resulting in different LDH release and caspase-3/7 activity in LNCap prostate cancer, which is responsible for necrosis and apoptosis, respectively; (**b**) Effects of saline, BA and hollow BNs-b spheres on mice preinjected with LNCap prostate cancer cells, respectively. (**c**) Percentage of mice without development of tumour over time after LNCap cancer cell injection (data are shown as mean±s.d., Kaplan–Meier log rank test, *P<0.05 vs saline group, *n*=4); (**d**) Quantitative analysis of the effects of different samples on tumour size (data are shown as mean±s.d., *t*-test, *P<0.05, *n*=4).

We investigated *in vivo* anticancer efficacy of BA and hollow BN spheres to suppress the tumour growth in mice with prostate cancers induced by the injection of LNCap prostate cancer cells through subcutaneously injected models first (Fig. 6). Saline group was used as the control. BN spheres and BA significantly suppress the prostate tumour growth compared with the control (Fig. 6c). Among all the groups, BNs-b is the most effective for controlling tumour progression. Thirty-three days after inoculation of LNCap cells, 50% of mice in the saline groups are free from tumour formation by visual inspection, compared with 75% in the BA, BNs-a and BNs-c groups and 100% in the BNs-b group. After 61 days, ratios of tumour-free mice for the saline, BA, BNs-a, BNs-b and BNs-c groups are 0, 50, 75, 100 and 25%, respectively. Finally, after 96 days, the ratios of tumour-free mice for the saline, BA, BNs-a, BNs-b and BNs-c groups are 0, 25, 50, 75 and 25%, respectively. Over the 3-month period, the average tumour volume increases to 827 mm^3^ in the saline group, compared with 2 mm^3^ in the BNs-b group and 287 mm^3^ in the BA group (Fig. 6d). It can be seen that BNs-b can inhibit tumour volume by about 99.75% compared with the control. The fact that the tumour growth is significantly suppressed in the BA group over the control group indicates that B can inhibit prostate cancer. The inhibitory effects on prostate tumour growth are further enhanced by the alternative use of BNs-a or -b sphere as a novel B carrier to realize the sustained release of B. However, BNs-c spheres exhibit much weaker inhibitory effects on prostate tumour growth owing to too high crystallinity associated with too low B release.

**Figure 7:**
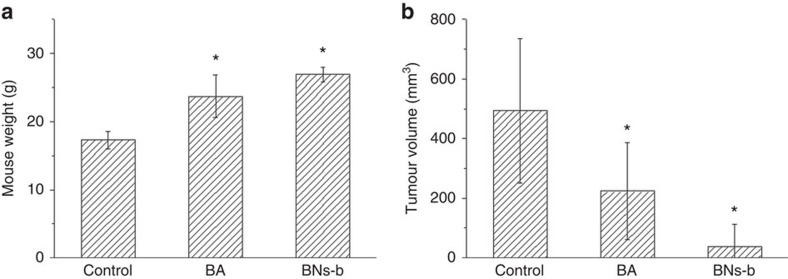
*In vivo* anticancer effects of BN spheres by using orthotopically injected models. (**a**) Mouse weight for different groups 12 weeks after LNCap cancer cell injection at the dose of 2 × 10^6^ cells per mouse (*n*=4); (**b**) quantitative analysis of the effects of different samples on tumour size at the end point (data are shown as mean±s.d., *t*-test, *P<0.05, *n*=4).

Then orthotopical tumour growth models in mouse injected with low dose of LNCap cells were used to further confirm *in vivo* anticancer efficacy of BA and hollow BN spheres to suppress the LNCap tumour occurrence and growth (Fig. 7). At the end point, 12 weeks later, the average mouse weight for the saline, BA and BNs-b groups is 17, 24 and 27 g, respectively, while the average tumour volume is in the following sequence: control group (494 mm^3^)>BA group (224 mm^3^)>BNs-b group (39 mm^3^). The orthotopical tumour growth models exhibit the same trends with subcutaneously injected models.

**Figure 8:**
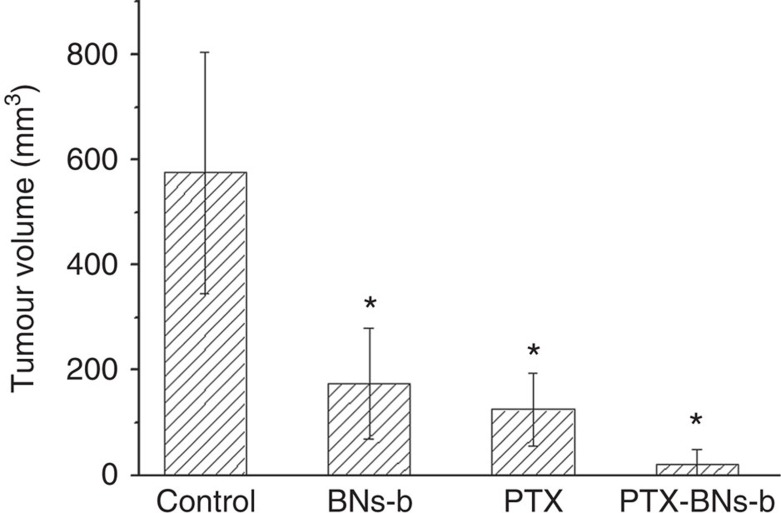
Comparison of cancer therapy efficacies using different combinations of BN spheres and PTX. Quantitative analysis of the tumour size for different groups 8 weeks after LNCap cancer cell injection at the dose of 5 × 10^6^ cells per mouse (data are shown as mean±s.d., *t*-test, *P<0.05, *n*=4).

Cancer therapy efficacies using hollow BN spheres, chemotherapy drug paclitaxel (PTX) and the PTX–hollow BN spheres complex were further evaluated in mice orthotopically injected with high dose of LNCap cells (Fig. 8). Both hollow BNs-b spheres and PTX drugs significantly inhibit the tumour growth compared with the control of saline group. Most importantly, the combination of hollow BNs-b spheres with PTX drugs demonstrates the cooperative and synergetic effects on the tumour suppression, as the PTX–hollow BN spheres complex group exhibits the minimum tumour volume among all the groups.

### *In vivo* safety and systemic biodistribution

Healthy C57/BL6 mice were intravenously administered via tail vein with 50 μg of BNs-b and then the blood biochemistry, haematology and biodistribution analysis were carried out. Various biochemistry parameters, such as blood urea nitrogen, creatinine, alanine aminotransferase, aspartate aminotransferase, alkaline phosphatase were tested (Supplementary Fig. 9A–E). The liver function markers (alanine aminotransferase, aspartate aminotransferase, alkaline phosphatase) and kidney function markers (creatinine, blood urea nitrogen) are only slightly varied and are still within the normal range, compared with the control. No obvious hepatic or renal toxicity is observed in treated mice. For the haematological analysis, the following important parameters, such as white blood cells, red blood cells, haemoglobin, haematocrit, mean cell volume, mean corpuscular hemoglobin, mean corpuscular hemoglobin concentration and platelet, were tested (Supplementary Fig. 10). All of the above parameters in the BNs-b treated groups appear to be normal compared with the control groups. As a whole, no obvious toxicity of BNs-b is noted from the blood biochemistry and haematological data.

Moreover, the biodistribution of BNs-b after intravenous injection into C57/BL6 mice was monitored after 1 h, 1 day, 3 days and 15 days (Supplementary Fig. 9F). During the whole period of time, no obvious signs of abnormal behaviour in eating, drinking and activity were documented. At a certain time, the mouse was killed, various organs and tissues were collected and the boron contents were measured by ICP. The results show that BNs-b distributes in many different organs and mainly accumulates in the reticuloendothelial system such as the liver and spleen. The distributed amounts in the different organs and tissues decrease with time owing to gradual degradation and clearance.

## Discussion

Challenges in applying nanomaterials in medicine are rapidly increasing and offer excellent prospects for the development of new non-invasive or minimally invasive strategies for the treatment of cancer^34^,^38^,^40^,^41^. Several kinds of nanoparticle systems for prostate cancer diagnosis and therapy^42^,^43^,^44^ have previously been developed to improve the efficacy of specific delivery and to decrease the incidence of serious side effects. However, in all cases, nanoparticles are used to act as the carrier for other chemotherapy drugs. Here we designed BN as a substantial anticancer nanomedicine that delivers and slowly releases B to play the important anticancer roles.

BN nanomaterials have recently attracted attentions in the biomedical field, for example, with respect to bone tissue engineering^45^, drug delivery^32^,^46^, boron neutron capture cancer therapy^47^, irreversible lethal electroporation cancer treatment^48^ and so on. In the previous study, surface-modified BN nanotubes^32^ and highly water-soluble porous BN nanomaterials^33^ were effectively loaded with doxorubicin and enhanced intracellular drug delivery into LNCap prostate cancer cells. Moreover, boron neutron capture cancer therapy is a targeted radiation therapy for cancer that significantly increases the therapeutic ratio relative to conventional radiotherapeutic modalities^49^. Boron-containing nanoparticles, such as BN nanotubes^47^, boron carbide^50^ and C_2_B_10_ carborane cage-attached carbon nanotubes^51^ have shown high concentration of boron atoms in tumour cells than in blood and other organs, which provided the targeted delivery of boron to tumour cells for an effective boron neutron capture under cancer therapy. However, compared with other nanomedicines, the exploration of BN nanomaterials for cancer diagonosis and therapy is just emerging. In this study, we have first demonstrated a cancer-therapeutic function in hollow BN nanomaterials.

Hollow BN spheres with controlled crystallinity were successfully fabricated using conventional CVD system and subsequent Ar treatment. The second-stage annealing process conducted in Ar rather than in NH_3_ atmosphere is the key point to form the hollow structures, which is quite different from the solid sphere in the previous report^39^. The CVD reaction of B(OMe)_3_ with ammonia initially results in the formation of the spherical precursor B(OMe)_3-x_H_3-x_N, which is a metastable intermediate phase. Moreover, the remnant oxygen impurity also has a crucial role in the resultant spherical morphology and accumulation of B-O in the core rather than in the sphere surface^39^. Thus, in the second-stage annealing process conducted in Ar, evaporation of B-O species causes voids in the obtained BN spheres. With the increase in temperature, the void size increases and shell thickness decreases.

Thus the control of B release was realized through adjusting the treatment temperature and the crystallinity of BN nanospheres. In this study, BNs nanospheres with different crystallinity and morphology were synthesized to act as a reservoir of B. B has a high affinity to oxygen and is present in aqueous solution, depending on pH, as either BA (B(OH)_3_) or borate (B(OH)_4_)^−^. As the pKa of the equilibrium between B(OH)_3_ and (B(OH)_4_)^−^ is 9.2, at the intracellular pH=7.4 free boron exists as the weak Lewis acid BA^22^. BN can be hydrolysed into BA and ammonia, which then transformed into ammonium borate hydrates^52^. An amorphous form is more soluble as compared with a crystalline form owing to the random configuration of atoms in an amorphous matter as compared with the ordered configuration in a crystalline matter. BNs-a treated at 900 °C has an amorphous nature and high surface energy, which facilitated dissolution of BN and the release of B. With the increase in treatment temperature for BNs-b to 1,025 °C, stability of the crystal lattice is gradually increased and the solubility of BN is gradually decreased. For BNs-c treated at 1,400 °C, the crystal structure is highly ordered having superb stability, which results in more energy required to break down a crystal lattice, low solubility of BN and a slow release of B.

Since recently, a mainstream medical path of boron has been focussed on drugs and surgical procedures as a means of therapy for prostate cancer^14^,^15^. The prostate cancer cell lines include LNCap, DU-145, PC-3 and so on. BA, the dominant form of boron in plasma, has a number of distinctive features that make it a promising pharmaceutical agent for cancer treatments. BA is a mild organic Lewis acid with structural features similar to carbon, allowing it to act as a competitive inhibitor for many carbon-containing substrates, such as kinds of enzymes^21^. This characteristic of BA makes it an effective inhibitor of enzymes, such as peptidases, proteases, proteasomes, arginase, nitric oxide synthase and transpeptidases^21^. BA in the blood lowers the risk of prostate cancer by inhibiting the NAD metabolite cADPR and reducing intracellular calcium signaling and storage^19^,^23^. At an appropriate concentration, BA selectively inhibits prostate cancer cell proliferation while allowing non-tumorigenic cells to grow^14^. BA decreases serum PSA levels by 88%, inhibits the LNCap tumour growth by 38% and reduces insulin-like growth factor-1 in nude mice injected with human LNCap prostate cancer cells^15^. Boron compounds inhibit the activity of serine protease, including PSA^16^, presumably by binding to its active site^53^. BA of 250μM or 1 mM causes a reduction in cell volume, F-actin-stained filopodia extending around the periphery of the cells and thus cell migration and invasion of human DU-145 prostate cancer cells in a dose-dependent manner^22^.

*In vitro* and *in vivo* experimental results confirm the advantage of the BN nanospheres as a delivery vehicle of B over BA to prevent prostate cancer and inhibit tumour growth. Although BA inhibits the prostate cancer cell activity in a dose-dependent manner, the effective dose (400 μM or 25 μg ml^−1^ ∼1,000 μM) *in vitro* is 40–100 times higher than average serum levels of BA in human^21^. The free B form, such as BA, will quickly excrete via urine with a half-life time of 24 h (ref. 54) and cannot take efficacy for a long time, which needs frequent and high-dose B administration that may result in drug resistance and side effects to healthy tissues. For instance, BA (1.7 mg B kg^−1^ day^−1^, equal to 42.5 μg B per mouse per day) was needed to administer every day by oral gavage to inhibit LNCap tumour growth in the mice by 38% (ref. 15). In contrast, in our study, the sustained release of B from BNs-b nanospheres obviously prevented LNCap tumour occurrence by 75% and inhibited LNCap tumour growth by 99.75%, when administrating them around the tumour sites at the dose of 30 μg BN per mouse once in 4 days for the initial 3 times and once in 7 days afterwards. Thus it is possible to achieve a high concentration of B in the tumour while avoiding accumulation of B in the normal tissues. Unexpectedly, hollow BN spheres with moderate crystallinity and B release speed effectively prevent prostate cancer and inhibit tumour growth.

The death mechanism study demonstrates that hollow BN spheres result in apoptosis and necrosis in prostate cancer cells, which corresponds to caspase-3/7 activity and LDH release, respectively (Figs 5 and 6a). Caspases are crucial mediators of programmed cell death (apoptosis). Among them, caspase-3/7 is a frequently activated death protease, catalysing the specific cleavage of many key cellular proteins. A key signature for necrotic cells is permeabilization of plasma membrane, which can be quantified in tissue culture settings by measuring the release of the intracellular enzyme LDH. For the androgen-sensitive LNCap prostate cancer cells, the hollow BNs-b spheres lead to a significantly higher fraction of apoptosis and necrosis than BA or the BNs-a spheres with low crystallinity. While for the androgen-independent DU145 prostate cancer cells, the hollow BNs-b spheres mainly result in a significant increase in apoptosis fraction. This trend corresponds to the LDH and caspase-3/7 assay. Totally, LNCap cells exhibit much higher LDH release than DU145 cells, and in turn, DU145 cells exhibit higher caspase-3/7 activity than LNCap cells.

Both LDH release and caspase-3/7 activity result from the synergetic effects of B release and BN nanoparticle-like morphology (Fig. 6a). As a whole, BN particle form has the leading role in LDH release, although the increase in dose of BA also results in the LDH release (Supplementary Figs 7 and 8). In general, LDH release is proportional to the amounts of nanoparticle. With an increase in crystallinity and decrease in solubility, BN spheres or BA cause the increase in LDH release in the sequence of BNs-c>BNs-b>BNs-a>BA>control. While for caspase-3/7 activity in prostate cancer cells, sustained B release and residual BN particle form make a coordination. The BNs-b sample with moderate B release and residual particle form with B active sites shows the highest caspase-3/7 activity.

The present hollow BN spheres hold promise in cancer therapy field. They show excellent water solubility and degradability, especially for those treated at low temperature, which is very important for the potent biomedical application. As shown in Fig. 8, both hollow BNs-b spheres and PTX drugs effectively suppress the tumour growth compared with the control group. Although the average tumour volume for hollow BNs-b group is slightly larger than that for PTX drugs, the hollow BN spheres are expected to decrease the medical cost and minimize the side effects compared with the high price and strong toxicity of PTX drugs. The particle size of about 200 nm for the as-prepared hollow BN spheres and its further decrease during degradation process provides opportunities for enhanced permeability and retention effects and thus passive targeting into tumour sites^30^. Moreover, the hollow BN spheres with hollow core within the spherical structures and moderate hydroxyl groups provide the possibility to load and protect chemotherapy drugs to realize the synergetic effects for cancer therapy. In this study, the combination of hollow BNs-b spheres with PTX drugs shows most effective antitumour efficacy among all the groups owing to the synergetic effects of hollow BN spheres and PTX drugs. In addition, BA was reported to help protect against genotoxicity and cytotoxicity in lymphocytes induced by PTX^55^.

Our results suggest that the hollow BN nanospheres could be used as therapeutic agents to inhibit the tumour recurrence in patients with prostate cancer, rather than for the prevention of prostate cancer in healthy individuals. Although preliminary experiments were carried out in orthotropic mouse models injected with prostate cancer cells lines to evaluate the antitumour efficacy of the nanospheres, it should be noted that these models do not fully mimic the human disease process and therefore are not suitable to assess the effects of boron compounds on the prevention of prostate cancer occurrence in healthy persons. To do so, a genetically engineered mouse models that mimic human disease development should be considered in future studies.

In summary, we have demonstrated an exciting new therapeutic property of hollow BN spheres with controlled release of B for prostate cancer treatment. Hollow BN spheres induce apoptosis and inhibit the proliferation for both the androgen-sensitive LNCap and androgen-independent DU145 prostate cancer cells. The mechanism study shows that they can result in the increase in caspase-3/7 activity and LDH release. *In vivo* assay demonstrates that hollow BN spheres significantly suppress the tumour occurrence and growth in male BALB/c-nu/nu mice models injected with LNCap prostate cancer cells.

## Methods

### Hollow BN spheres synthesis

Hollow BN spheres were prepared based on a traditional CVD system while making some modifications of our previously reported method^39^, in which the second-stage annealing process was conducted in Ar rather than in NH_3_ atmosphere. Briefly, trimethoxyborane (B(OMe)_3_) solution was bubbled into a quartz tube in a horizontal tube furnace by a nitrogen flow, which was heated to 980 °C. Ammonia was also fed separately and simultaneously into the chamber. The optimal gas flow rates were 300 sccm for N_2_ and 500 sccm for NH_3_ in the present work. After the CVD reactions, BN sphere precursor was collected from the inner wall of the quartz tube. To obtain hollow BN nanomaterials, the obtained precursor was further annealed at 900, 1,025 and 1,400 °C in Ar atmosphere for 4 h to improve the crystallinity. The obtained powders were designated as BNs-a, -b and -c, respectively.

### Characterizations of hollow BN spheres

The morphology, structure and chemical compositions were characterized by using a transmission electron microscope (JEOL JEM-3000F) operated at 300 kV, Fourier transform infrared spectroscopy (Thermo Nicolet 4700) and X-ray photoelectron spectroscopy (Alpha 110-mm analyser XPS version; Thermo Fisher Scientific, Chiyoda-ku, Tokyo, Japan). The phase composition and the degree of crystallinity were analysed by X-ray diffractometry employing CuKα X-ray at 40 kV and 40 mA using a powder X-ray diffractometer (Rigaku Ultima III, Japan). The boron release from BN nanospheres contained in a bag of dialysis membrane in an acetate buffer (pH=4.6), a tris-HCl buffer (pH=7.4) or a carbonate buffer (pH=9.0) at a BN-to-buffer ratio of 25, 50, 125, 250 or 500 μg ml^−1^ was quantitatively analysed after incubation at different temperatures (25, 50, 100 or 120 °C) using an inductively coupled plasma atomic emission spectrometer (ICP: SPS7800, Seiko Instruments, Japan). Particle size and zeta potential were analysed using a Delta Nano C Particle Analyzer (Beckman Coulter) by dispersing particles in water and PBS buffer, respectively.

### Cell viability assay

LNCap prostate cancerous cells (RIKEN Bio Resource Center, Japan) with a cell density about 0.5 × 10^4^ cells cm^−2^ were cultured in 48-well plates containing RPMI1640 medium supplemented with 10% fetal bovine serum, 100 units ml^−1^ penicillin and 100 μg ml^−1^ streptomycin at 37 °C in humidified air containing 5% CO_2_. DU145 prostate cancerous cells (RIKEN Bio Resource Center, Japan) were cultured under the same conditions except without the use of antibiotics. After overnight, hollow BN suspensions were added into the above wells at a final concentration of 0, 5 and 25 μg ml^−1^, respectively. BA containing almost equivalent amount of B was also added as a control. The cell viability was checked using a Cell Counting Kit-8 Kit (Dojindo Molecular Technologies, Japan) in accordance with the manufacturer's instructions. Cell viability was analysed by a Student's *t*-test. Differences were considered significant at *P*<0.05.

### Detection of apoptosis and necrosis

The TACS Annexin V kits (Trevigen) was used to check the apoptosis and necrosis of LNCap or DU145 prostate cancerous cells. Apoptosis and necrosis were analysed by double staining with Annexin V-FITC (fluorescein isothiocyanate) and propidium iodide (PI). Annexin V bound to apoptosis cells with exposed phosphatidylserines, while PI labelled necrotic cells with membrane damage. Normal healthy cells were double negative. Early apoptotic cells were positive for Annexin V staining but negative for PI staining, while late apoptotic cells were double positive. Cells stained with PI only were considered to be necrotic. LNCap or DU145 cells were incubated with hollow BN suspensions in six-well plates at concentrations of 5 and 25 μg ml^−1^ for 3 or 6 days. After incubation, adherent cells were harvested by trypsinization and all floating and adherent cells were centrifuged and washed once with cold PBS. Annexin V-FITC/PI incubation reagent in binding buffer was added to the cells and incubated in the dark for 15 min at room temperature. Then 400 μl of binding buffer was added to the cells suspension, and the cells were analysed by using a Sony spectral cell analyser LE-SP6800A. Single-factor analysis of variance (ANOVA) was conducted to identify significant differences among the groups. When significant differences were found, Tukey's *post hoc* multiple-comparison test was used to determine the significant differences between the mean values of the groups. Differences were considered significant at *P*<0.05.

### Caspase-Glo 3/7 assay

Caspase-3/7 activities were measured in cells treated with hollow BN nanospheres using the Caspase-Glo 3/7 Assay Kits (Promega) according to the manufacturer's protocol. Briefly, 100 μl LNCap cells (5 × 10^4^ cells cm^−2^) were seeded into each well of a 96-well culture plate and incubated at 37 °C overnight. Then 100 μl of hollow BN suspensions were added to the well to get the final concentration of 5 μg ml^−1^ of BN or BA containing almost equivalent B followed by incubation for 16 h. Single-factor ANOVA was conducted to identify significant differences among the groups. When significant differences were found, Tukey's *post hoc* multiple-comparison test was used to determine the significant differences between the mean values of the groups. Differences were considered significant at *P*<0.05.

### LDH assay

LDH cytotoxicity caused by hollow BN spheres was measured by a detection kit (Takara Bio Inc. Japan). Briefly, 100 μl LNCap cells (5 × 10^4^ cells cm^−2^) were seeded into each well of a 96-well culture plate and incubated at 37 °C overnight. Then 100 μl of hollow BN suspensions at various concentrations were added to the well to get the final concentrations of 1, 5 and 25 μg ml^−1^ of BN or BA containing almost equivalent B followed by incubation for 16 h. The microplate was centrifuged to get the supernatant. LDH cytotoxicity was checked by using the above supernatant with a reaction time about 12 min in accordance with the manufacturer's instructions. The Triton X-100 (1%) was used as a positive control in the assay. Single-factor ANOVA was conducted to identify significant differences among the groups. When significant differences were found, Tukey's *post hoc* multiple-comparison test was used to determine the significant differences between the mean values of the groups. Differences were considered significant at *P*<0.05.

### *In vivo* antitumour experiment

Male BALB/c-nu/nu mice (Charles river, Japan) were used in this study. Human LNCap prostate cancer cells were collected from exponentially growing cultures with 90% confluent.

For subcutaneous tumour growth models, 20 mice were divided randomly into 5 groups. Collected LNCap cells were mixed with Matrigel 1:1 by volume and kept on ice until use. A total amount of 8 × 10^6^ LNCap cells were injected subcutaneously into the left flank of the mice. Four days later, mice were administered subcutaneously approximately 0.5–1 cm apart from the tumour site with saline, hollow BN suspension in saline (BNs-a, BNs-b, BNs-c, 30 μg, respectively) or BA containing almost equivalent B in saline, once in 4 days for the initial 3 times and once in 7 days afterwards before killing. Tumour diameter was measured using calipers at a certain time interval. Once the tumour diameter reached 2 cm, the experiment was discontinued to limit the tumour burden in mouse. Percentage of mice without development of tumours was analysed by a Kaplan–Meier log-rank test. Tumour volume was analysed by Student's *t*-test. Differences were considered significant at *P*<0.05.

For orthotopical tumour growth models, 12 mice were divided randomly into 3 groups. A transverse incision was made in the lower abdomen. The bladder and seminal vesicles were delivered through the incision to expose the prostate. When the prostate was identified, a total amount of 2 × 10^6^ cells suspended in 20 μl of HEPES buffer were carefully injected under the prostatic capsule. The incision was closed using a suture of 4-0 silk. Mice were administered intravenously with PEG-saline (1 mg ml^−1^), hollow BNs-b (30 μg) suspension in PEG-saline or BA containing almost equivalent B in PEG-saline, once in 1 day for the initial 3 times and once in 4 days for the subsequent 6 times. Then, 12 weeks later, body weights were measured and a transverse incision was made in the lower abdomen to monitor the tumour size. Tumour volume and body weights were analysed by a Student's *t*-test. Differences were considered significant at *P*<0.05.

In addition, orthotopical tumour growth models were also used to check and compare the treatment efficacy of hollow BN spheres, clinically used chemotherapy drug (PTX) and the PTX–hollow BN spheres complex. The saline group was used as a control. Sixteen mice were divided randomly into four groups. A total amount of 5 × 10^6^ cells suspended in 30 μl of HEPES buffer were carefully injected under the prostatic capsule. Mice were administered intravenously with saline, hollow BNs-b (30 μg) suspension in PEG-saline, PTX (15 μg) in saline or the PTX–hollow BN spheres complex in PEG-saline, once in 1 day for the initial 3 times and once in 3 days for the subsequent 3 times. Then, 8 weeks later, a transverse incision was made in the lower abdomen to monitor the tumour size. Tumour volume was analysed by a Student's *t*-test. Differences were considered significant at *P*<0.05.

### *In vivo* safety and biodistribution

To examine the *in vivo* safety, six C57/BL6 mice were divided randomly into two groups and administered BNs-b (50 μg in PEG-saline) or saline intravenously via tail-vein injection. The acute toxicology, such as blood biochemistry and haematology analysis, was measured 3 days later. The results were analysed by a Student's *t*-test. Differences were considered significant at *P*<0.05. In addition, 12 C57/BL6 mice were divided randomly into 4 groups and administered BNs-b (50 μg in PEG-saline) intravenously via tail-vein injection. At several fixed time points (1 h, 1 day, 3 days, 15 days), the tissues, such as heart, liver, spleen, lung, kidney and stomach, were discretized. The tissue distribution of BNs-b particles was semiquantified by hydrothermal digestion in 1 M NaOH solution. The double-blind experiments were carried out.

All the animal experiments were permitted by the Ethical Committee of the National Institute for Materials Science (NIMS), Japan. All the animal experiments and feeding were carried out in accordance with the guidelines of the Ethical Committee of NIMS, Japan.

### Data availability

The data that support the findings of this study are available from the authors upon reasonable request.

## Additional information

**How to cite this article:** Li, X. *et al*. Hollow boron nitride nanospheres as boron reservoir for prostate cancer treatment. *Nat. Commun.* **8**:13936 doi: 10.1038/ncomms13936 (2017).

**Publisher's note:** Springer Nature remains neutral with regard to jurisdictional claims in published maps and institutional affiliations.

## Supplementary Material

Supplementary Information Supplementary Figures.
