# Supplementary material for: Hollow boron nitride nanospheres as boron reservoir for prostate cancer treatment
Source: Nat Commun. 2017 Jan 6;8:13936. doi: 10.1038/ncomms13936 (PMC5228389; doi:10.1038/ncomms13936)
Supplement: Supplementary Information — Supplementary Figures. [file ncomms13936-s1.pdf]

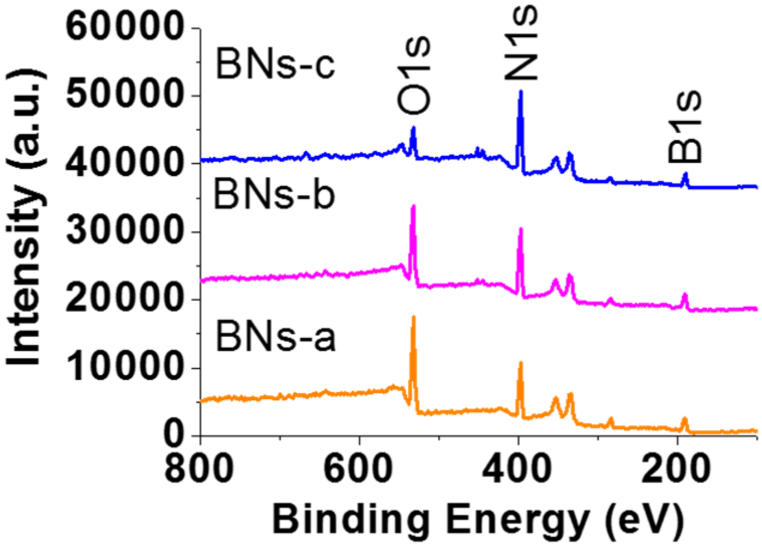

Supplementary Figure 1. Wide range XPS spectra of BN spheres (BNs-a, BNs-b and BNs-c).

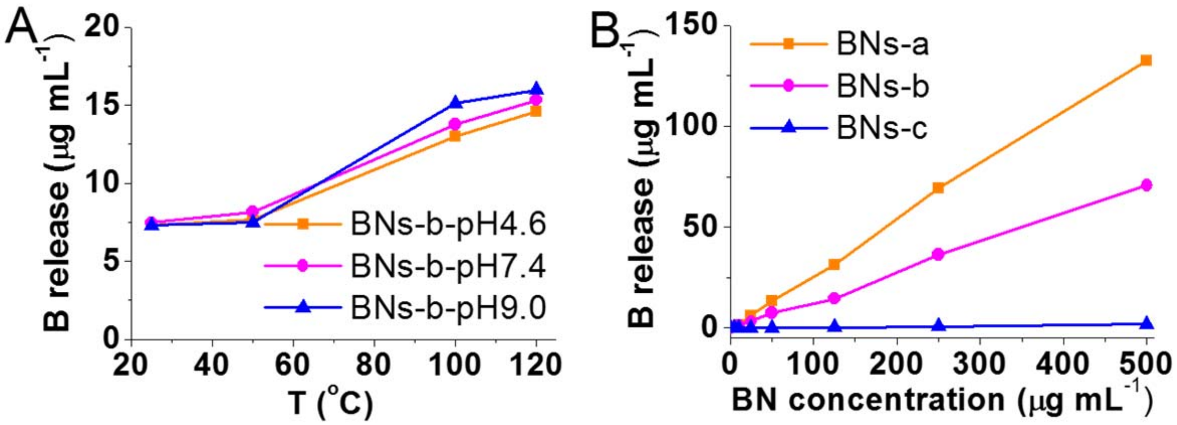

Supplementary Figure 2. B release in different buffer (pH=4.6, 7.4, 9.0) for BNs-b at different temperatures and pH values after 1 day (a); B release in culture medium for BNs-a, BNs-b and BNs-c with different initial concentrations at room temperature after 5 days (b).

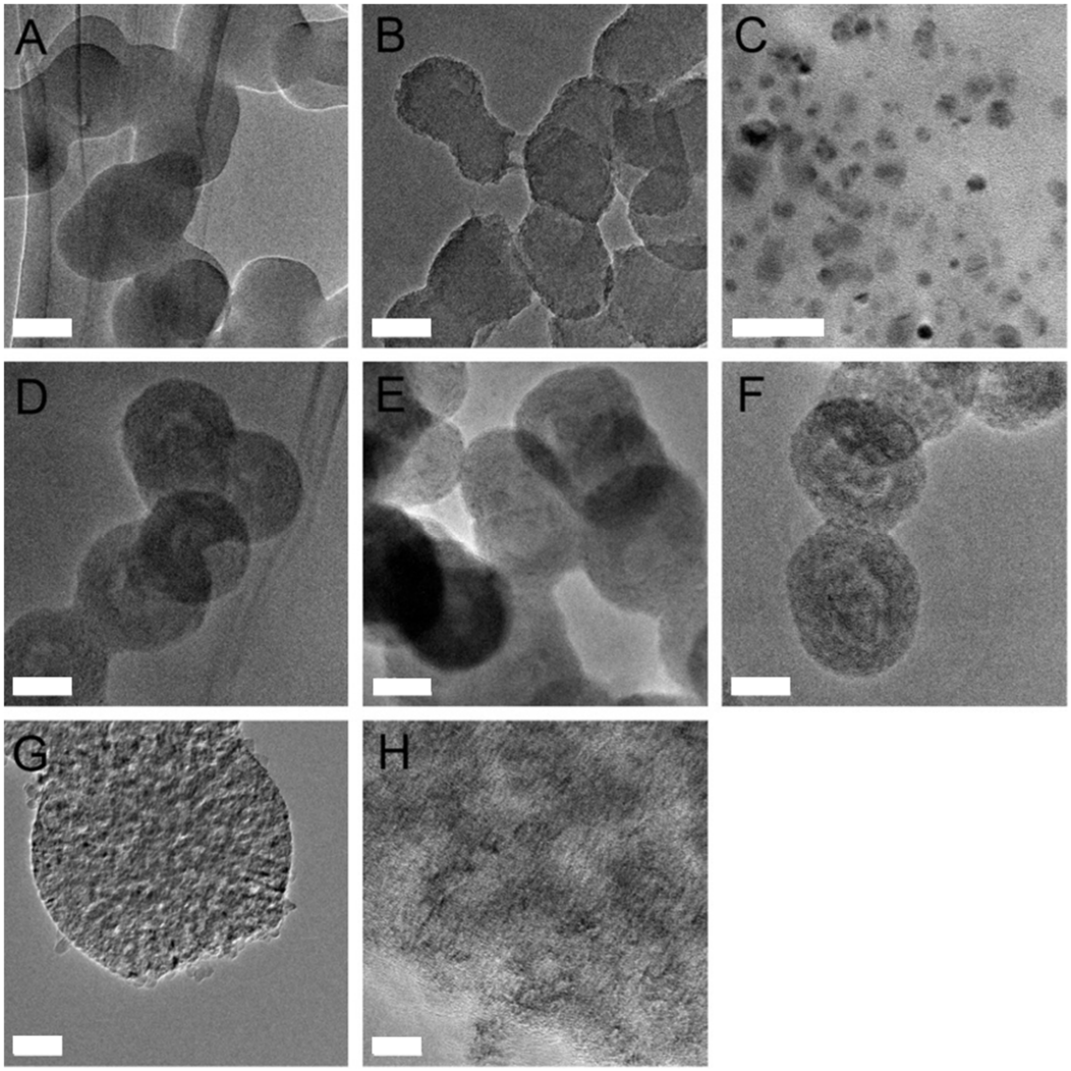

Supplementary Figure 3. Dynamic studies of structural evolution of BN spheres: BNs-a in Tris-HCl buffer after 2 hours (A, scale bar 100nm), 1 day (B, scale bar 100nm) and 3 days (C, scale bar 50nm); BNs-b in Tris-HCl buffer after 2 hrs (D, scale bar 100nm), 1 day (E, scale bar 100nm), 3 days (F, scale bar 100nm) and 10 days (G, scale bar 50nm; H, scale bar 10nm).

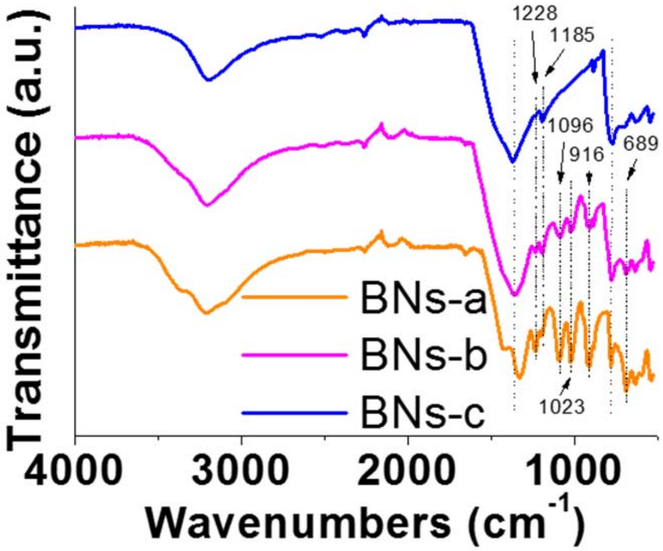

Supplementary Figure 4. FTIR spectra of BN nanospheres after immersion in ultrapure water at 40°C for 3 days.

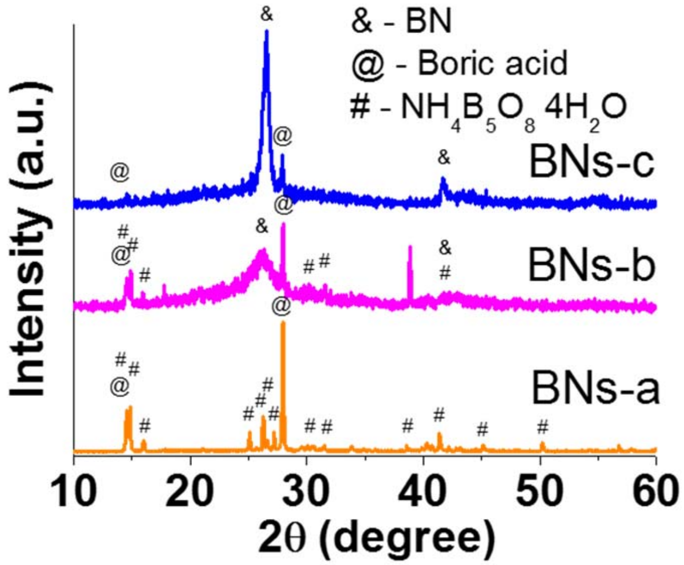

Supplementary Figure 5. WAXRD patterns of BN nanospheres after immersion in ultrapure water at 40°C for 3 days.

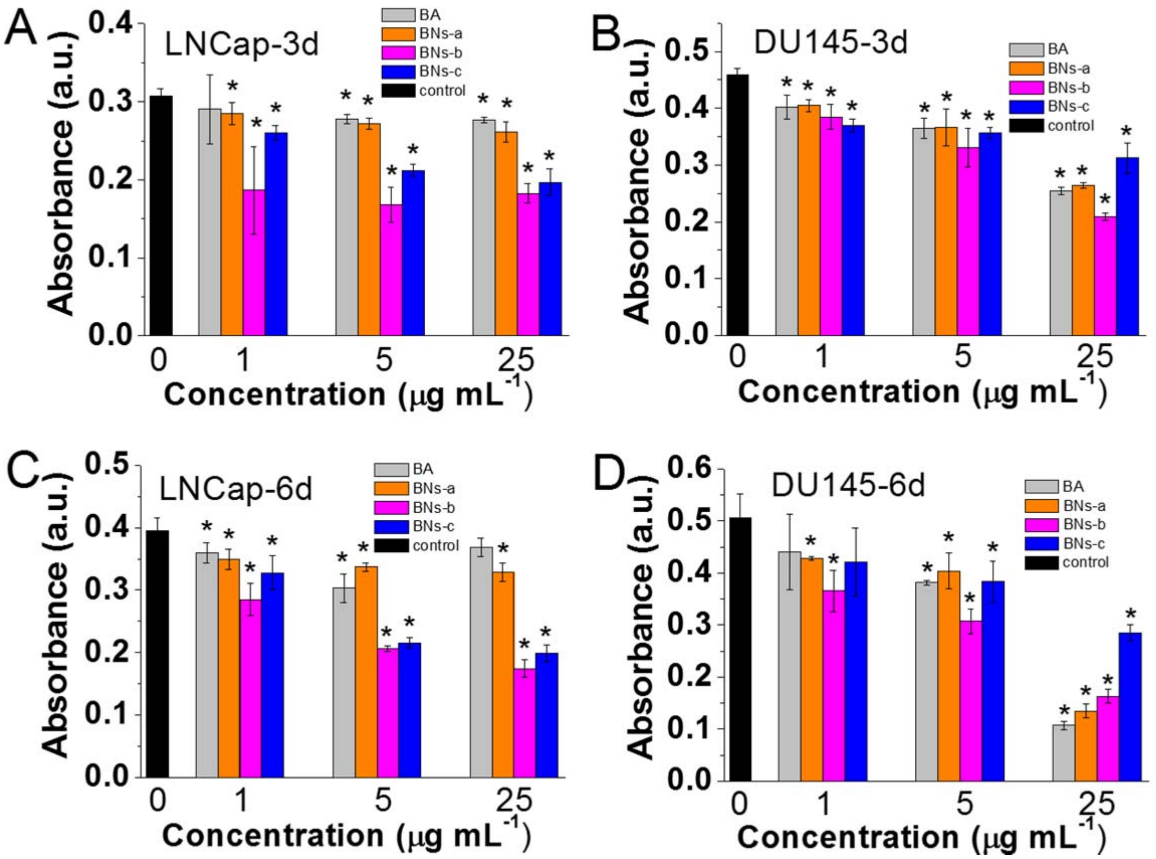

Supplementary Figure 6. LNCap and DU145 cells viability after 3 or 6 days culture with BNs or BA (at the same B concentration) (Data is shown as mean ± s.d., t-test, *p<0.05 vs control, n=4).

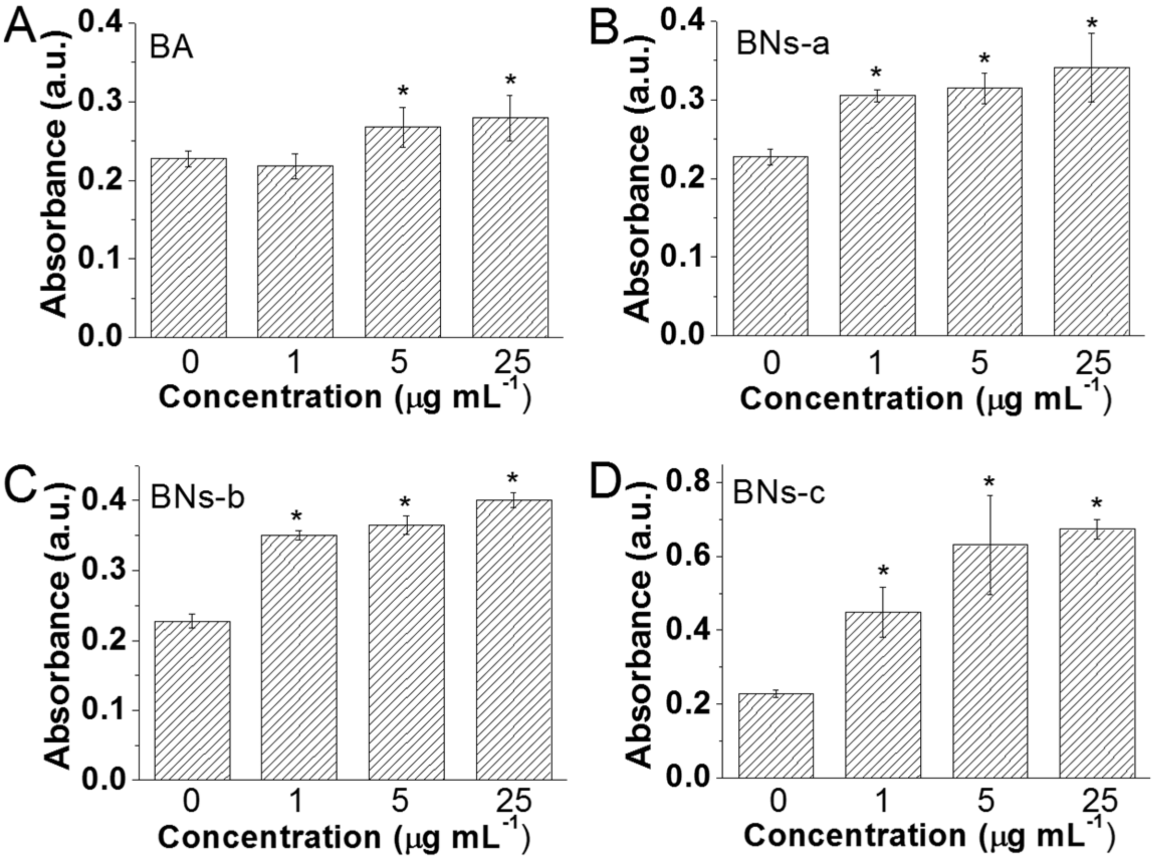

Supplementary Figure 7. LDH cytotoxicity for DU145 prostate cancer cells after 16 hrs with an increased amount of BNs or BA (at the same B concentration) (Data is shown as mean±s.d., t-test, *p<0.05 vs control, n=4).

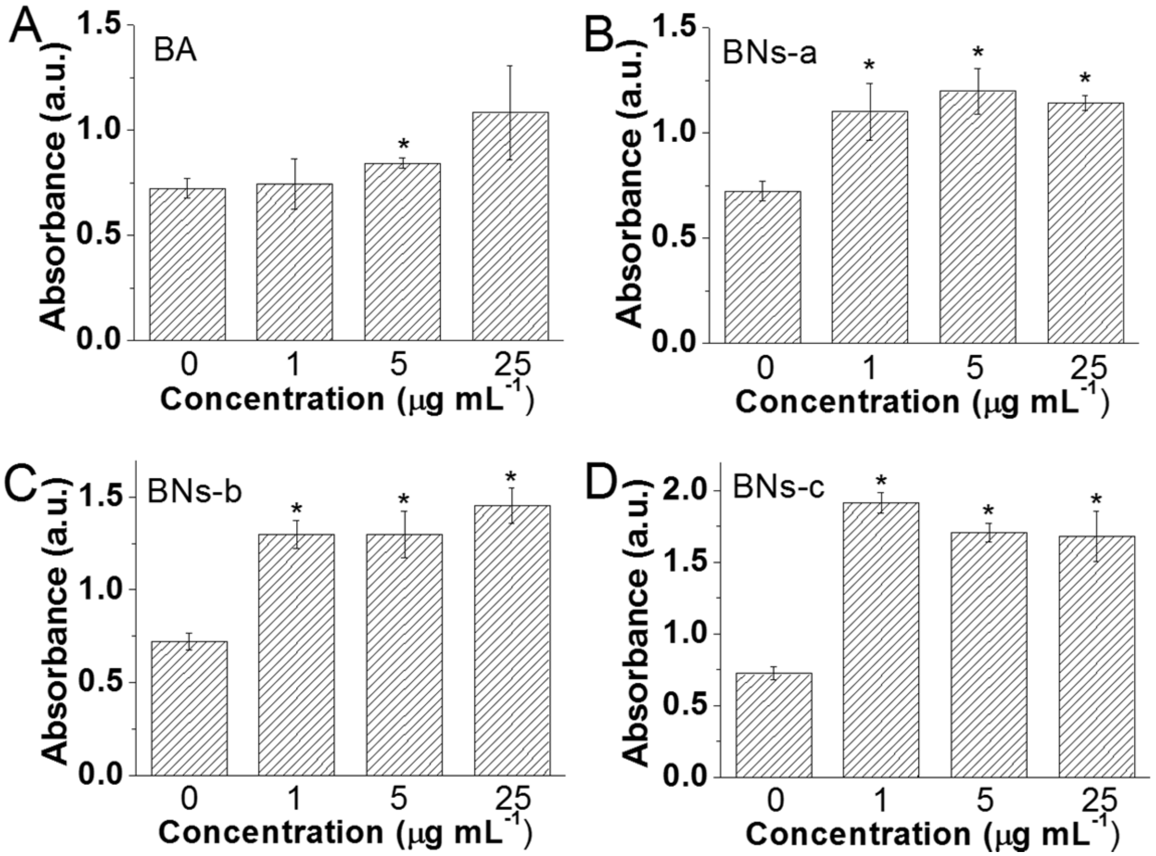

Supplementary Figure 8. LDH cytotoxicity for LNCap prostate cancer cells after 16 hrs with an increased amount of BNs or BA (at the same B concentration) (Data is shown as mean ± s.d., t-test, $^*p<0.05$ vs control, n=4).

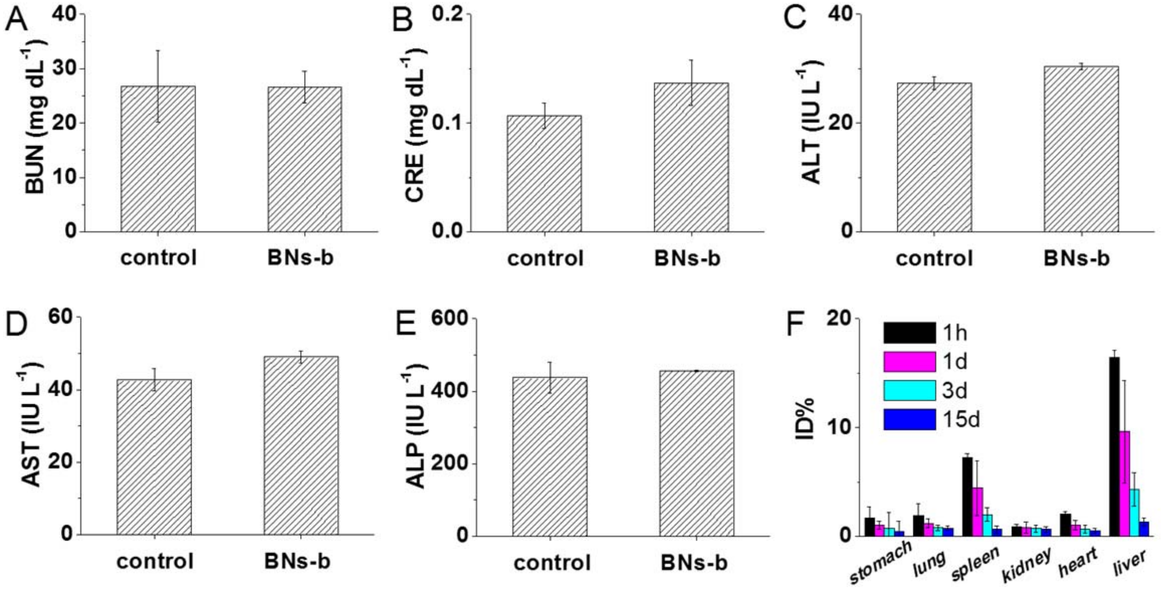

Supplementary Figure 9. Blood biochemistry analysis (a-e) and biodistribution (f) of mice after intravenous administration with BNs-b (Data is shown as mean ± s.d., n=3).

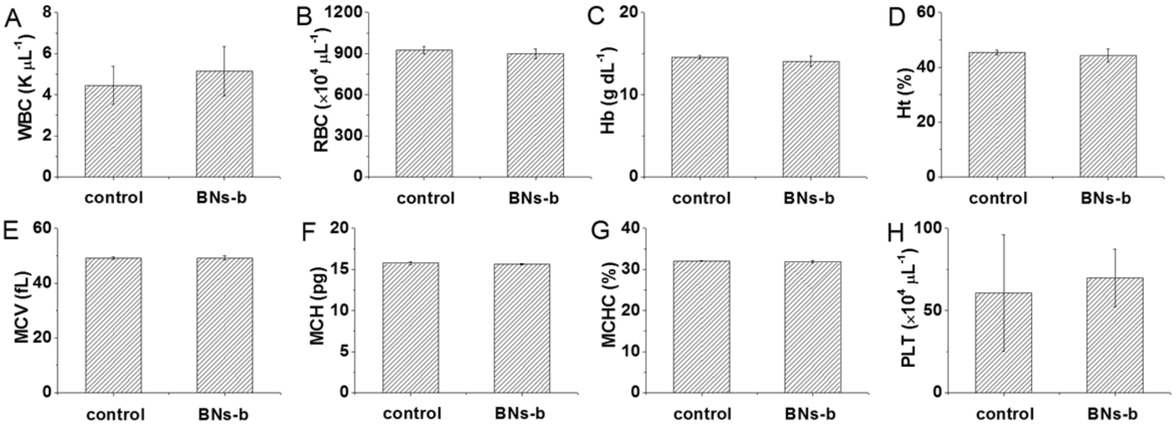

Supplementary Figure 10. Blood hematological analysis of mice after intravenous administration with BNs-b (a-h) (Data is shown as mean ± s.d., n=2).
